# An Ovine Model of Awake Veno-Arterial Extracorporeal Membrane Oxygenation

**DOI:** 10.3389/fvets.2021.809487

**Published:** 2021-12-23

**Authors:** Jiachen Qi, Sizhe Gao, Gang Liu, Shujie Yan, Min Zhang, Weidong Yan, Qiaoni Zhang, Yuan Teng, Jian Wang, Chun Zhou, Qian Wang, Bingyang Ji

**Affiliations:** ^1^Department of Cardiopulmonary Bypass, State Key Laboratory of Cardiovascular Medicine, Fuwai Hospital, National Center for Cardiovascular Disease, Chinese Academy of Medical Science and Peking Union Medical College, Beijing, China; ^2^Beijing Key Laboratory of Pre-clinical Research and Evaluation for Cardiovascular Implant Materials, State Key Laboratory of Cardiovascular Medicine, Fuwai Hospital, National Center for Cardiovascular Disease, Chinese Academy of Medical Science and Peking Union Medical College, Beijing, China

**Keywords:** extracorporeal membrane oxygenation (ECMO), translational large animal model, critical care, cardiopulmonary support, perioperative management

## Abstract

**Background:** Large animal models are developed to help understand physiology and explore clinical translational significance in the continuous development of veno-arterial extracorporeal membrane oxygenation (VA-ECMO) technology. The purpose of this study was to investigate the establishment methods and management strategies in an ovine model of VA-ECMO.

**Methods:** Seven sheep underwent VA-ECMO support for 7 days by cannulation *via* the right jugular vein and artery. The animals were transferred into the monitoring cages after surgery and were kept awake after anesthesia recovery. The hydraulic parameters of ECMO, basic hemodynamics, mental state, and fed state of sheep were observed in real time. Blood gas analysis and activated clotting time (ACT) were tested every 6 h, while the complete blood count, blood chemistry, and coagulation tests were monitored every day. Sheep were euthanized after 7 days. Necropsy was performed and the main organs were removed for histopathological evaluation.

**Results:** Five sheep survived and successfully weaned from ECMO. Two sheep died within 24–48 h of ECMO support. One animal died of fungal pneumonia caused by reflux aspiration, and the other died of hemorrhagic shock caused by bleeding at the left jugular artery cannulation site used for hemodynamic monitoring. During the experiment, the hemodynamics of the five sheep were stable. The animals stayed awake and freely ate hay and feed pellets and drank water. With no need for additional nutrition support or transfusion, the hemoglobin concentration and platelet count were in the normal reference range. The ECMO flow remained stable and the oxygenation performance of the oxygenator was satisfactory. No major adverse pathological injury occurred.

**Conclusions:** The perioperative management strategies and animal care are the key points of the VA-ECMO model in conscious sheep. This model could be a platform for further research of disease animal models, pathophysiology exploration, and new equipment verification.

## Introduction

Veno-arterial extracorporeal membrane oxygenation (VA-ECMO) is used as a rescue therapy for patients with severe cardiopulmonary failure, and it can provide a bridge to recovery of the natural organs or transplantation (1, 2). Basic research in animals plays an important role in the continuous development of VA-ECMO technology. Small animal models are mainly used for the study of molecular biological mechanisms, whereas large animal models are developed to help understand physiology and explore clinical translational significance.

Previous studies on ECMO in large animals were mostly in the acute stage, and the survival time of large animals was within 24 h (3). Some studies have reported the use of ECMO on awake, non-intubated, spontaneously breathing large animals, as this strategy offers several benefits over mechanical ventilation (4–6). However, most awake ECMO methods focused on veno-venous modality (7, 8), and a few cases of awake VA-ECMO have been reported, especially support for up to 7 days (9). As there are some differences between large animals and clinical situations, optimized establishment methods and perioperative management strategies are still lacking in large animal models supported by VA-ECMO. Based on our previous studies on the survival rat model of cardiopulmonary support (10, 11), we aimed to establish an awake ovine VA-ECMO model that could run for 7 days and to investigate the establishment method and perioperative management using this model.

## Materials and Methods

### Animals and Preparation

This experimental protocol was approved by the Institutional Animal Care and Use Committee (IACUC) of Fuwai Hospital [no. 0101-2-20-HX(X)], and all procedures were in accordance with the Guide for the Care and Use of Laboratory Animals published by the National Institutes of Health, USA (publication no. 86-23, revised 1996). The experiment was conducted at Beijing Key Laboratory of Pre-clinical Research and Evaluation for Cardiovascular Implant Materials, Animal Experimental Center of Fuwai Hospital (registration no. CNAS LA0009). The experimental animals were all healthy sheep with qualified quarantine provided by the Animal Experimental Center of Fuwai Hospital. During the whole experimental process, we strictly followed the ARRIVE (Animal Research: Reporting of *in vivo* Experiments) guidelines for pre-clinical animal studies.

Seven healthy 12- to 24-month-old adult male sheep (Small Tailed Han sheep, weight = 54–63 kg; Beijing Jinyutongfeng Trading Co., Ltd., Beijing, China) received VA-ECMO implantation (Table 1). Sheep received 24-h cage-side care and were monitored by a veterinarian according to the animal management protocol. The pre-operative fasting time was 48 h and the water deprivation time was 12 h.

**Table 1:** Detailed characteristics of experimental sheep.

**Sheep number**	**Gender**	**Weight (kg)**	**Duration (days)**	**Termination**	**Cannulation site**
S2020-014	M	58	7	Scheduled	Vjr (24-Fr)–Ajr (18-Fr)
S2020-038	M	57	7	Scheduled	Vjr (24-Fr)–Ajr (18-Fr)
S2020-040	M	54	7	Scheduled	Vjr (24-Fr)–Ajr (18-Fr)
S2020-041	M	55	7	Scheduled	Vjr (24-Fr)–Ajr (18-Fr)
S2020-043	M	56	7	Scheduled	Vjr (24-Fr)–Ajr (18-Fr)
S2020-042	M	63	1	Accidental death	Vjr (24-Fr)–Ajr (18-Fr)
S2020-016	M	56	2	Accidental death	Vjr (24-Fr)–Ajr (18-Fr)

*M, male; Vjr, right jugular vein; Ajr, right jugular artery*.

### Device

The VA-ECMO circuit consisted of an arterial cannula (18-Fr; Edwards Lifesciences, Irvine, CA, USA), a venous cannula (24-Fr; Edwards Lifesciences), a centrifugal pump drive and console (OASSIST STM001; Jiangsu STMed Technologies Co., Suzhou, China), a disposable centrifugal pump (STM CP-24 I; Jiangsu STMed Technologies Co.), and an oxygenator kit (Hilite7000LT; XENIOS, Heilbronn, Germany, or BE-PLS 2050, Maquet, Rastatt, Germany). The ECMO system was primed with 800 ml Ringer's lactate solution.

### Surgical Procedure

Anesthesia was induced with propofol (3–5 mg/kg) through the auricular vein. Electrocardiogram monitoring was connected and basic hemodynamics were recorded. Tracheal intubation was conducted with a single-lumen endotracheal tube (#10). Then, the intubation was connected for mechanical ventilation. The ventilation mode was the volume control mode, the tidal volume was 8–10 ml/kg, the respiratory rate was 12–20 breaths/min, and the fraction of inspiration oxygen (FiO_2_) was 60%. General anesthesia was maintained through isoflurane inhalation (2–3%) and propofol injection (8–10 mg kg^−1^ h^−1^). Intraoperative blood pressure and heart rate were maintained within ±20% of their baseline values, and the partial pressure of end tidal carbon dioxide (PaCO_2_) was maintained at 35–45 mmHg.

After the induction of anesthesia, the experimental sheep were immobilized on the operating table in the supine position. A single-lumen central venous catheter (18-G) was placed in the left jugular artery (arterial line) and a three-lumen central venous catheter (7-Fr) placed in the left jugular vein (venous line). These two lines were used for hemodynamic monitoring, intravenous fluids, drug injection, and blood collection.

Systemic anticoagulation was performed with a bolus of heparin (120 IU/kg) after the right jugular artery and vein were exposed. The target activated clotting time (ACT) of the cannulation was higher than 250 s. The arterial cannula (18-Fr) was inserted through the right common carotid artery, with the cannula descending 10–15 cm, while the venous cannula (24-Fr) was inserted through the right external jugular vein to the right atrium.

**Figure 1:**
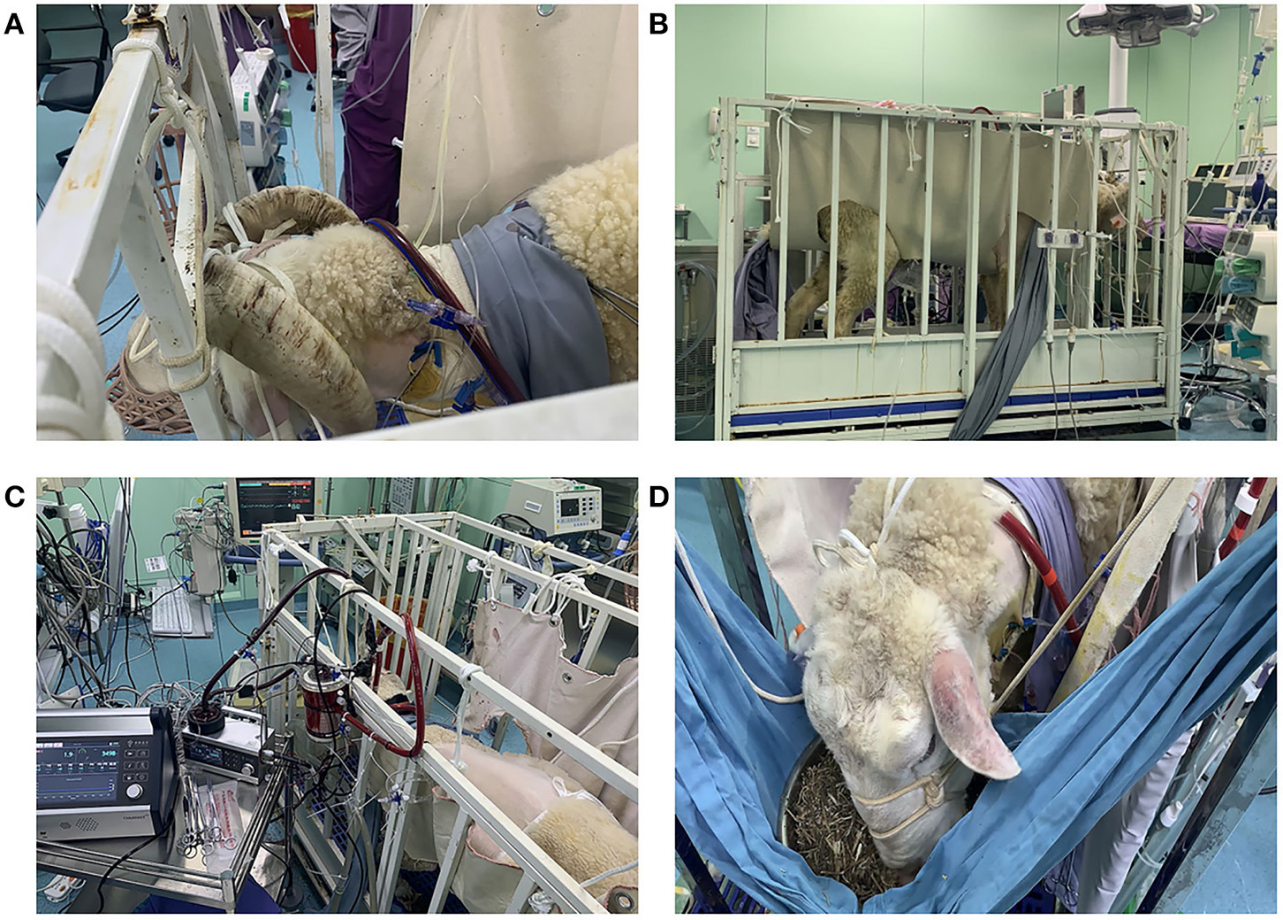
**(A)** Fixation of the cannula around the neck (from *right* to *left side*). **(B)** The sheep were moved into a metabolic cage and properly restrained after the operation. **(C,D)** The sheep could eat and move freely within a certain range in the monitoring cage. They were provided with appropriate amount of hay, pellets, and water every day.

The centrifugal pump and a membrane oxygenator were connected and primed. The venous cannula was connected to the pump inlet, while the arterial cannula was connected to the oxygenator outlet. Extracorporeal circulation was started with a pump flow of 2.0–2.5 L/min and a rotational speed of 3,200–3,500 rpm. Then, the incision was sutured, the cannula was fixed securely, and the circuit line was half looped around the neck to avoid displacement and kinking (Figure 1A).

The sheep were moved into a metabolic cage and properly restrained (Figure 1B) after the operation was completed and vital signs were stable. Special attention was paid to the fixation of the head and shoulder of the sheep to prevent the cannula from dislocation or kinking. Then, the depth of anesthesia was gradually reduced. After the sheep recovered to spontaneous respiration and the blood gas analysis was stable, the endotracheal tube was extubated.

### Post-surgical Care, Monitoring, and Data Collection

Extracorporeal circulation was maintained with a target pump flow of 2.0 L/min (30 ml kg^−1^ min^−1^) and pump speed around 3,500 rpm during the experimental period. The sweep gas flow of the oxygenator was 1.0–1.5 L/min at a concentration of 50–80%. Dynamic adjustment was made according to the blood gas analysis, venous oxygen saturation (SvO_2_), and arterial oxygen saturation (SaO_2_). Heparin was infused continuously to maintain ACT in the range of 220–250 s. In the first 24 h after surgery, flurbiprofen axetil (1–2 mg/kg) and dexmedetomidine (0.2–0.3 μg kg^−1^ h^−1^) were administered intravenously.

After the surgery, the sheep stayed awake in the monitoring cage. The animals freely ate hay and feed pellets and drank water. The daily volume load was maintained at a positive balance around 1,000 ml, while the central venous pressure was maintained at 5–12 cm H_2_O. Intravenous infusion was adjusted according to the intake, urine volume, blood pressure, and mental state. After operation, antibiotics were used daily to prevent infection (cefuroxime sodium, 1.5 g, i.v., b.i.d.). The incision was disinfected daily, and the infection and bleeding signs were observed at the same time.

Basic hemodynamics (including heart rate and mean arterial pressure) of experimental sheep and the ECMO hydraulic parameters (including speed, flow rate, pre-pump pressure, post-pump pressure, and post-oxygenator pressure) were monitored in real time. Blood gas analysis (Abbott i-STAT1; Abbott Point of Care Inc., Princeton, NJ, USA) and ACT (Hemochron Signature Elite; Hemochron, Bedford, MA, USA) were tested every 6 h. Complete blood count (ADVIA 2120i; Siemens Healthcare, Erlangen, Germany), free hemoglobin (fHb) (DiaSpect T Low Hemoglobin Analyzer; DiaSpect Medical GmbH, Sailauf, Germany), blood chemistry (Catalyst One; IDEXX Laboratories, Inc., Westbrook, MA, USA), and coagulation test (Fully Automated Coagulation Analyzer SF-8050; Beijing Succeeder Technology Inc., Beijing) were monitored daily. After 168 h (7 days), the ECMO system was removed and the sheep were euthanized by venous administration of potassium chloride (100 mg/kg) under the sedation of propofol (20 mg/kg). Necropsy was conducted by two pathologists to visualize the cannula position *in vivo* and to examine cannulation-related injury and thrombus formation. The main organs (heart, lung, kidney, liver, brain, and intestine) were removed for further histopathological evaluation.

### Histological Analysis

Specimens of the main organs and blood vessels obtained through pathological anatomy were cut into small pieces and fixed with 10% neutral fumarin fix solution (Yili Fine Chemical Co., Ltd., Beijing, China). The fixed tissues were embedded in paraffin and hematoxylin–eosin staining (hematoxylin; Yili Fine Chemical Co., Ltd.; eosin; Sigma-Aldrich Trading Co., Ltd., Shanghai, China) was performed on 5-μm-thick samples. Histological analysis was performed under a light microscope and evaluated by two pathologists.

### Data Analysis

Data were analyzed using SPSS software (version 26.0; IBM Corp., Chicago, IL, USA) and GraphPad Prism 8 (version 8.4.0; San Diego, CA, USA). Continuous variables were presented as the mean ± standard deviation (SD), and significant differences at various time points were determined with one-way analysis of variance with *post-hoc* multiple comparisons. A *p* < 0.05 was considered statistically significant.

## Results

Seven sheep survived surgery and recovered from anesthesia. Five sheep survived and successfully weaned from ECMO. Two sheep died within 24–48 h of ECMO support. One animal (no. S2020-016) suffered low partial pressure of arterial oxygen (PaO_2_, 62 mmHg), while the oxygenation performance of the oxygenator decreased (PaO_2_ after the oxygenator was 85 mmHg) after 6 h of ECMO support. After continuous liquid therapy and application of vasoactive drugs (epinephrine and norepinephrine), the blood pressure of the sheep continued to decrease. The sheep finally died of respiratory and circulatory failure after 48 h of ECMO support. The other animal developed neck swelling after 8 h of ECMO support, and then the hemoglobin decreased continuously. Although we performed a second operation to explore the bleeding point and remove the hematoma, the sheep still died of hemorrhagic shock after 24 h of ECMO support. These two sheep were not included in our subsequent data and histological analysis.

### Hemodynamics and ECMO Performance

**Figure 2:**
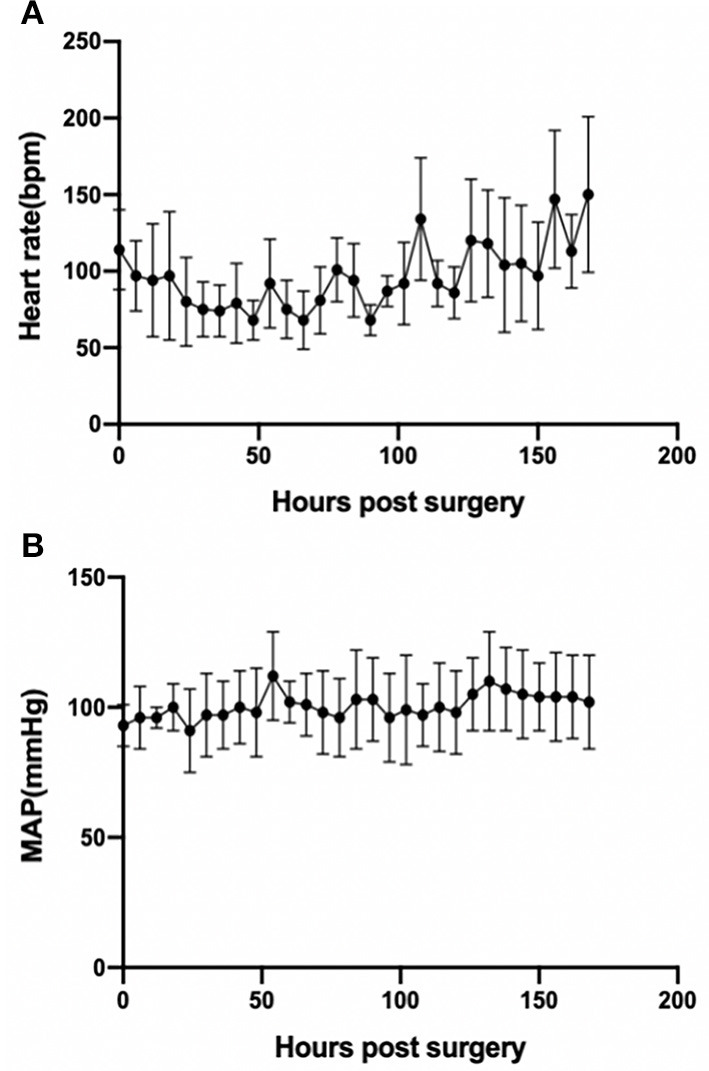
Basic hemodynamics as assessed by heart rate (*p* < 0.001) **(A)** and MAP (*p* > 0.05) **(B)** during the experimental period. *Error bars* show the standard deviation. *MAP*, mean arterial pressure.

**Figure 3:**
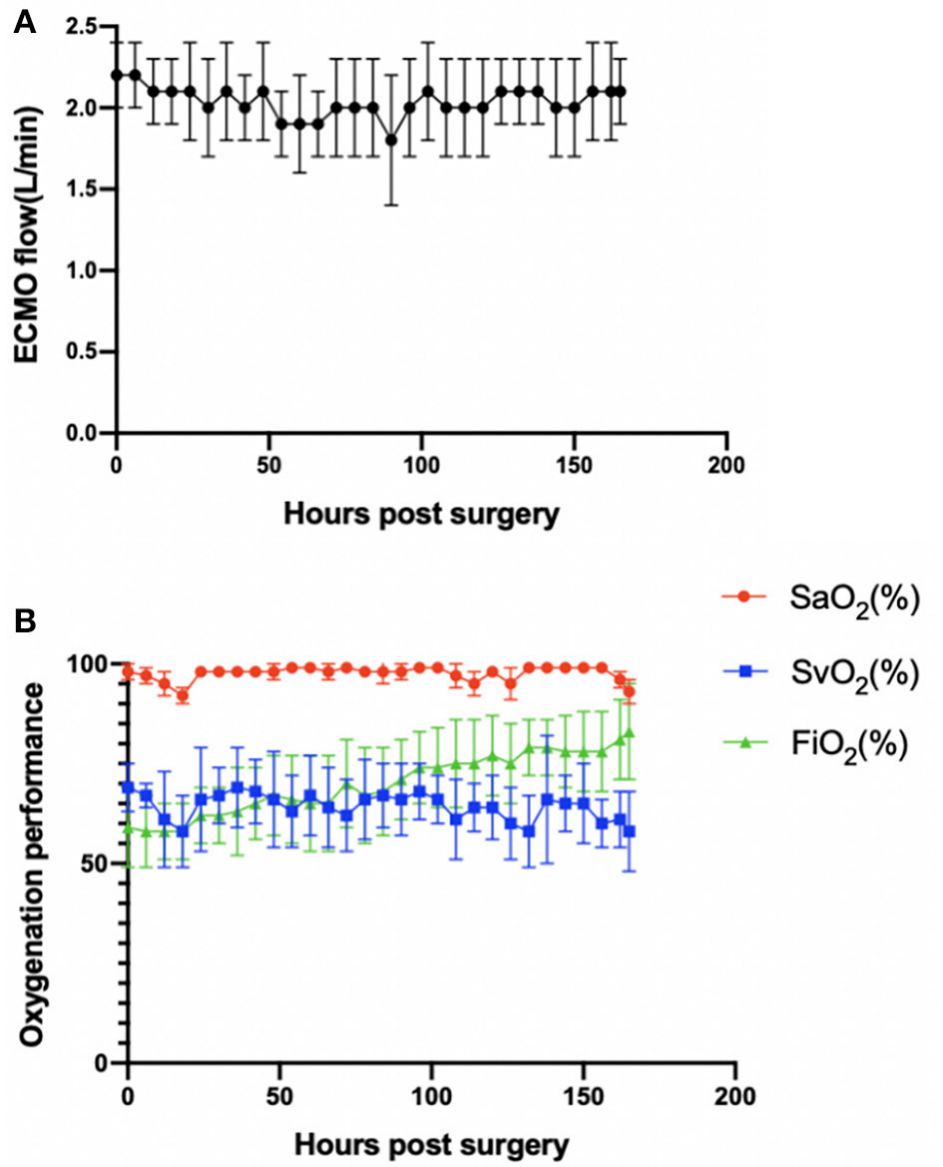
**(A)** ECMO flow was stable (*p* > 0.05). **(B)** Oxygenation performance of the oxygenator was good (*p* > 0.05). *Error bars* show the standard deviation. *ECMO*, extracorporeal membrane oxygenation; *SaO*_2_, oxygen saturation after the oxygenator; *SvO*_2_, oxygen saturation before the oxygenator; *FiO*_2_, fraction of inspiration oxygen.

The animals stayed awake and freely ate hay and feed pellets and drank water (Figures 1C,D). Hemodynamics were in the normal physiological range throughout the experimental period (Figure 2), with no need of inotropic medicine. During the experiment, the ECMO flow was stable (*p* > 0.05; Figure 3A) and the oxygenation performance of the oxygenator was good (*p* > 0.05; Figure 3B).

### Hematology, Hemolysis, and Coagulation Results

Table 2 summarizes the changes of the main hematology, hemolysis, and coagulation results. Considering the basic physiological values of hematology in sheep (12), the hemoglobin levels remained stable with no blood transfusion (*p* > 0.05), and the platelet counts were within the physiologic range, although slightly decreased, and then increased after surgery. White blood cell (WBC) counts increased at 48, 72, 144, and 168 h after surgery, while high-sensitivity C-reactive protein (hsCRP) was still within normal ranges (*p* > 0.05). The fHb concentration was kept at a low level after surgery (*p* > 0.05). The ACT was maintained at the target level by dynamically adjusting the heparin dosage.

**Table 2:** Main hematology, hemolysis, coagulation, and biochemical results.

**Variable^a^**	**Pre-surgical**	**Hours after surgery**							
		**6 h**	**24 h**	**48 h**	**72 h**	**96 h**	**120 h**	**144 h**	**168 h**
**Main hematology, hemolysis and coagulation results**									
Hb (g/L)	102 ± 15	104 ± 22	112 ± 9	106 ± 10	103 ± 11	113 ± 10	101 ± 12	102 ± 8	102 ± 13
PLT (×10^9^/L)	162 ± 85	166 ± 82	138 ± 58	130 ± 53	142 ± 52	182 ± 98	222 ± 191	275 ± 200^*^	276 ± 157^*^
WBC (×10^9^/L)	6.63 ± 0.81	7.20 ± 4.23	7.20 ± 4.23	14.66 ± 5.73^*^	9.17 ± 1.18^*^	6.88 ± 3.11	7.96 ± 1.77	9.54 ± 2.58^*^	14.23 ± 6.34^*^
hsCRP (mg/L)	0.28 ± 0.10	0.19 ± 0.13	0.21 ± 0.12	0.12 ± 0.14	0.19 ± 0.10	0.19 ± 0.14	0.06 ± 0.03	0.10 ± 0.10	0.18 ± 0.09
fHb (g/L)	0.28 ± 0.07	0.12 ± 0.16	0.24 ± 0.05	0.20 ± 0.11	0.14 ± 0.12	0.24 ± 0.05	0.14 ± 0.12	0.20 ± 0.00	0.16 ± 0.08
ACT (s)	165 ± 20	252 ± 40	273 ± 24	233 ± 27	226 ± 32	249 ± 22	231 ± 13	242 ± 14	230 ± 9
**Main biochemical indicators**									
TP (g/L)	68.8 ± 10.2	64.2 ± 7.9	65.2 ± 6.8	65.8 ± 6.0	67.8 ± 5.9	69.4 ± 5.3	71.7 ± 2.7	69.8 ± 5.3	73.7 ± 7.2
ALB (g/L)	27.0 ± 3.4	25.4 ± 1.7	25.8 ± 2.6	26.3 ± 2.5	26.4 ± 2.3	27.0 ± 2.3	27.6 ± 2.7	26.6 ± 3.2	27.8 ± 3.3
Cr (μmol/L)	135.7 ± 15.4	134.5 ± 25.1	128.1 ± 20.5	109.2 ± 16.2	114.6 ± 20.2	110.8 ± 15.5	110.6 ± 14.3	115.4 ± 24.8	109.7 ± 27.6

*Hb, hemoglobin; PLT, platelet; WBC, white blood cells; hsCRP, high-sensitivity C-reactive protein; fHb, plasma-free hemoglobin; ACT, activated clotting time; TP, total protein; ALB, albumin; Cr, creatinine*.

* *p < 0.05 (compared with pre-surgical baseline)*.

a *Data are shown as the mean ± SD*.

### Blood Gas Parameters, Blood Chemistry, and Fluid Volume

**Figure 4:**
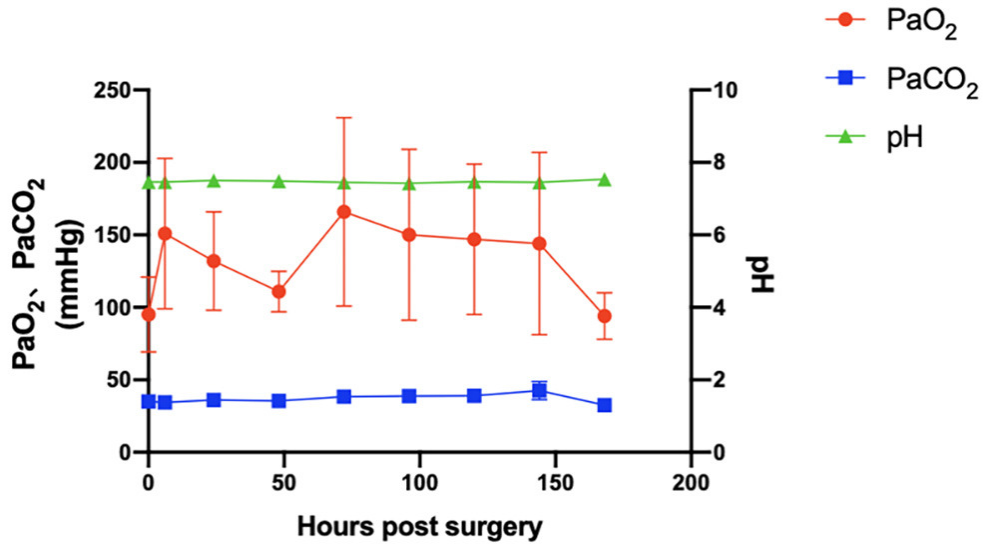
The main blood gas parameters of arterial blood pH, PaO_2_, and PaCO_2_ (*p* > 0.05) were maintained at normal levels. *Error bars* show the standard deviation. *PaO*_2_, partial pressure of arterial oxygen; *PaCO*_2_, partial pressure of arterial carbon dioxide.

The variety of main blood gas parameters is shown in Figure 4. Arterial blood pH, PaO_2_, and PaCO_2_ were maintained at relatively normal levels. Important biochemical indicators are also summarized in Table 2. The levels of total protein (TP) and albumin (ALB) were relatively stable (*p* > 0.05). The creatinine levels during VV-ECMO support and after weaning were lower than those of the baseline (*p* > 0.05). The daily volume load was maintained at a positive balance (1,096 ± 654 ml/day).

### Pathological Analysis

**Figure 5:**
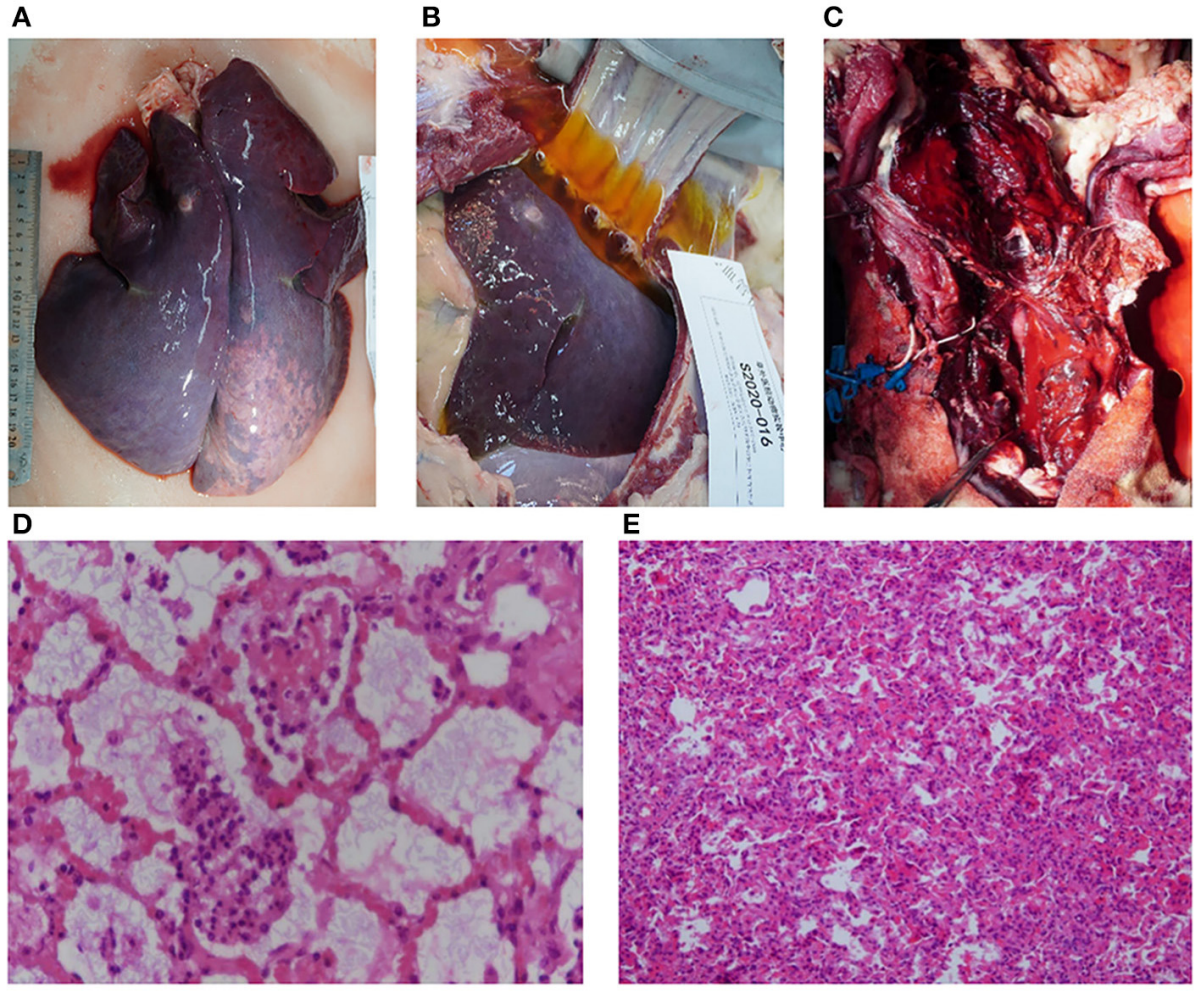
Pathological evaluation of dead animals. **(A)** Pulmonary edema, congestion, and consolidation were observed in both lungs (animal no. S2020-016). **(B)** A large amount of pleural effusion was observed (animal no. S2020-016). **(C)** Extensive hemorrhage and hematoma formation around the neck (animal no. S2020-042). **(D)** Inflammatory cells and fungal hyphae could be seen in the alveoli and bronchium under a light microscope (stained with hematoxylin and eosin, ×100 magnification) (animal no. S2020-016). **(E)** Atelectasis caused by hemothorax was observed under a light microscope (stained with hematoxylin and eosin, ×200 magnification) (animal no. S2020-042).

Two animals died within 24–48 h of ECMO support. According to the necropsy, one sheep died of respiratory and circulatory failure caused by fungal pneumonia within 48 h (no. S2020-016). Pulmonary edema, congestion, and consolidation occurred in both lungs accompanied by a large amount of pleural effusion (Figures 5A,B). Food fibers could be seen in the small airway, while a large number of fungal hyphae with suppurative inflammation in the alveoli were observed (Figure 5D). The other sheep died in the first 24 h of hemorrhagic shock caused by bleeding at the left jugular artery cannulation site, which was used for hemodynamic monitoring (no. S2020-042). Gross anatomy showed massive acute bleeding (mainly blood clots) in the neck and superior mediastinum on the opposite side (left) of the ECMO cannulation (Figure 5C). At the same time, hemothorax led to atelectasis (Figure 5E). There was an obvious edema and a small amount of bleeding at the cannulation site of the right jugular artery and vein.

**Figure 6:**
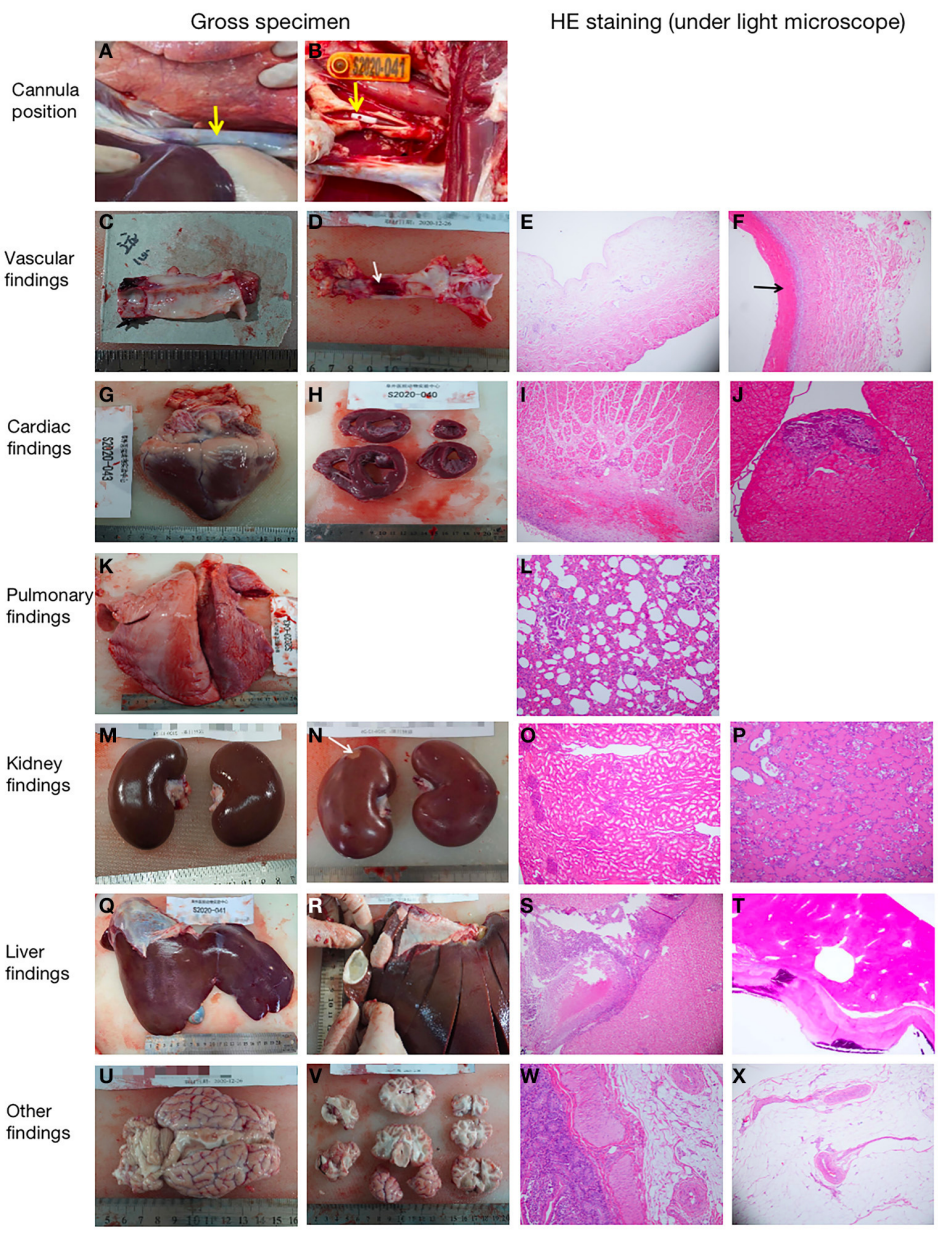
Representative pathological pictures. **(A)** The internal thoracic segment of the jugular vein cannula was observed *in situ*. The superior vena cava was not dissected (as shown by the *arrow*), and there was no cannulation displacement (animal no. S2020-038). **(B)** The tip of the cannula (as shown by the *arrow*) could be seen in the jugular artery (animal no. S2020-041). **(C)** Normal gross specimen of the right jugular intima (animal no. S2020-040). **(D)** Thrombus (as shown by the *arrow*) was seen in the middle intima of the right jugular vein (animal no. S2020-038). **(E)** The normal jugular vein was observed under a light microscope [stained with hematoxylin and eosin (HE), ×10 magnification] (animal no. S2020-014). **(F)** There was a small amount of thrombosis in the jugular vein at the cannulation site (as shown by the *arrow*) (HE, ×100 magnification) (animal no. S2020-038). **(G,H)** Normal gross specimens of the heart (animal nos. S2020-043 and S2020-040). **(I)** Normal myocardial tissue of the right atrium (HE, ×10 magnification) (animal no. S2020-038). **(J)** Small myocardial calcification (HE, ×20 magnification) (animal no. S2020-041). **(K)** Normal lung specimen (animal no. S2020-043). **(L)** Normal lung tissue (HE, ×20 magnification) (animal no. S2020-038). **(M)** Gross specimen of normal kidney (animal no. S2020-043). **(N)** Gross specimen of small renal infarction (as shown by the *arrow*) (animal no. S2020-038). **(O)** Normal kidney tissue (HE, ×10 magnification) (animal no. S2020-043). **(P)** Small infarct (HE, ×20 magnification) was observed in the kidney (animal no. S2020-038). **(Q)** Normal liver specimen (animal no. S2020-041). **(R)** Cyst was found in the liver (animal no. S2020-040). **(S)** Normal liver tissue (HE, ×10 magnification) (animal no. S2020-038). **(T)** Spontaneous cyst with calcification (HE, ×20 magnification) was found in the liver, (animal no. S2020-040). **(U,V)** Gross specimens of the brain. There was no hemorrhage or infarction (animal no. S2020-038). **(W)** Normal intestine tissue (HE, ×20 magnification) (animal no. S2020-043). **(X)** Normal mesentery tissue (HE, ×10 magnification) (animal no. S2020-014).

Five sheep survived and were successfully weaned from ECMO. Pathological anatomy showed that the position of the cannula was correct and there were no subcutaneous hematoma or other bleeding signs (Table 3; Figure 6). Thrombosis at the cannulation site occurred in three cases (3/5 cases; Table 3), but there was no vascular occlusion or stenosis. According to the histopathological evaluation, the organ infarction rate was low (1/5 cases; Table 3), and the infarct focal size was small (<5% surface areas, with no obvious clinical effect). Focal lymphocytic myocarditis near the epicardium was observed under a light microscope in one sheep (no. S2020-014; Table 3; Figure 6), but the size of the lesion was small (<5% surface areas) and there was no obvious clinical effect. Multiple small myocardial calcifications were observed under a light microscope in one sheep (no. S2020-041; Table 3; Figure 6), but were of no clinical significance. In addition, no major adverse pathological injury occurred.

**Table 3:** Main pathological findings.

**Sheep number**	**Cannulation position**	**Vascular injury at cannulation site**	**Cardiac findings**	**Pulmonary changes**	**Other changes**
S2020-014	Correct	None	Myocarditis	None	None
S2020-038	Correct	Thrombosis	None	None	Small focal renal infarction
S2020-040	Correct	None	None	None	Hepatic cyst
S2020-041	Correct	Thrombosis	Small calcification	None	None
S2020-043	Correct	Thrombosis	None	None	None

## Discussion

We established an awake VA-ECMO ovine model that achieved both respiratory and circulatory support for 7 days. By using the portable OASSIST ECMO system that has shown satisfactory safety and biocompatibility during the 7-day pre-clinical evaluation (13), 5 of the 7 sheep survived and were successfully weaned from ECMO. During the experiment, hemodynamics were in the normal physiological range, and no serious bleeding or coagulation events occurred. The animals obtained adequate nutrition from normal eating and maintained a satisfactory hemoglobin level, with no need of additional artificial nutritional support or blood transfusion. The ECMO flow remained stable, plasma fHb was maintained at a low level, and the oxygenation performance of the oxygenator was satisfactory. No major adverse pathological injury occurred.

In this study, extracorporeal circulation was maintained with a target rotational speed of 3,200–3,500 rpm and a pump flow of 2.0 L/min. Although the JACC Scientific Expert Panel stated that the flow of VA-ECMO could reach 4–6 L/min (50–70 ml kg^−1^ min^−1^) when supporting human patients with heart failure (14), a target pump flow of 2.0 L/min could satisfy the hemodynamic needs in our healthy sheep model with a functional native heart. As the healthy sheep has a functional native heart, a higher pump speed will increase the pre-pump pressure, resulting in blood damage. Besides, Iizuka et al. tested a novel centrifugal pump (CAPIOX SL) in chronic large animal (healthy sheep) experiments, with target pump flow of 2.0–3.0 L/min during the experimental period (9). Akiyama et al. also evaluated an ultra-compact durable ECMO system in healthy sheep, and the flow rate ranged from 2.2 ± 0.7 to 2.5 ± 0.1 L/min under a pump speed higher than 4,000 rpm (15). In our experiment, the speed was set at 3,200–3,500 rpm. The ECMO flow ranged from 1.8 ± 0.1 to 2.4 ± 0.14 L/min. This range can provide sufficient hemodynamic support comparable to that in previous animal studies. Further research on establishing a large animal disease model should dynamically adjust the target rotational speed and flow rate of VA-ECMO according to the specific situation.

At the beginning of our experiment, two sheep died of fungal pneumonia or hemorrhagic shock within 24–48 h after ECMO support. According to the necropsy, sheep no. S2020-016 died of respiratory and circulatory failure caused by fungal pneumonia within 48 h. Food fibers could be seen in the small airway, while a large number of fungal hyphae with suppurative inflammation in the alveoli were also observed. Therefore, we inferred that the fungal pneumonia was caused by reflux aspiration. At the same time, the anesthesia induction and the endotracheal intubation process of the experimental sheep were smooth, and oxygenation was normal during the maintenance of anesthesia. Therefore, we considered that it is highly possible that the reflux aspiration occurred in the period of anesthesia recovery (recovery to spontaneous respiration) after ECMO implantation. During the recovery period, the sheep did not fully recover muscle strength and manifested active cough and swallowing reflex, resulting in gastroesophageal reflux. To avoid the occurrence of reflux aspiration, the sheep should be fasted for at least 48 h and the endotracheal tube should be extubated when the sheep recovered spontaneous breathing, swallowing reflex, or airway protective reflex. Besides, we should pay more attention to cannulation. Effective hemostasis is required during cannulation, and observation for serious bleeding or thrombotic events is also needed during ECMO support. In addition, these adverse events indicate that the cannulation approach, perioperative management, and animal care are the key to the success of this model.

Sheep can be used to establish an awake ECMO model because they are docile and more conducive to perioperative management than are other large animals such as pigs and oxen. Different from the femoro-femoral cannulation approach often used in adult VA-ECMO patients, the femoral artery and vein of sheep are relatively thin, while the neck vessels are thick, and mobility is relatively fixed. In order to ensure the target flow and flow stability, we selected the neck vessels of sheep as the cannulation vessel. Besides, the upper-body cannulation approach is often preferred when mobility is of sufficiently high priority (16). In our model, the common carotid artery and external jugular vein were chosen to conduct the autonomous activity. The advantage of ambulation was obvious. Firstly, sheep breathed and coughed spontaneously, which might reduce pulmonary infection. Secondly, no intubation allowed independent feed for sheep to get adequate nutrition, contributing to appropriate hemoglobin, TP and ALB levels, and no need for blood transfusion. However, caring conscious and ambulatory sheep supported by ECMO met some difficulties. We paid attention to avoid the occurrence of reflux aspiration, as mentioned before. To ensure stable ECMO flow and avoid cannula dislocation or kinking, the cannula was fixed securely while the line was half looped around the neck. Special attention was paid to the fixation of the head and shoulder of the sheep using a “sling” (Figure 1B) to prevent the cannula from kinking.

Anticoagulant management is an important part of the perioperative management. In our preliminary experiment, the ACT was set at 180–220 s according to clinical situations and previous studies in sheep (7–9, 17, 18). However, fibrinogen accumulated and thrombus formed in the oxygenator within 48 h, indicating that sheep needed higher anticoagulant conditions. Therefore, we adjusted the target ACT to 220–250 s, while there was no serious bleeding or coagulation events occurred. The ACT should be monitored every 6 h throughout the experiment to detect the potential risk of heparin insufficiency or overdose.

Some retrospective studies have reported that fluid overload commonly occur in patients supported with ECMO (19). Progressive fluid overload during ECMO is associated with acute kidney injury, higher mortality, prolonged mechanical ventilation, and ECMO duration (20). Therefore, the daily fluid balance of the experimental sheep should be focused on. Unlike in clinical practice where patients tend to have cardiac failure, a negative fluid balance does not need to be controlled as healthy sheep have normal cardiac function. At the same time, excessive fluid negative balance can affect the performance of ECMO and lead to blood damage. Considering the insensible water loss (such as salivation) and the reduced food intake of sheep during ECMO support, the daily volume was maintained at positive balance around 1,000 ml (1,096 ± 654 ml/day). Intravenous infusion was adjusted properly according to the fluid balance. The creatinine levels during ECMO support were lower than those at baseline, and no pathological changes were found in the kidney, indicating that the fluid management was appropriate and the renal function was maintained within the normal range.

One advantage of this study is that it is the first to focus on the perioperative management and animal care in large animal models of awake VA-ECMO. Further studies should be conducted based on this model. Firstly, the interaction between cardiopulmonary support and natural perfusion should be explored. Besides, detailed hemodynamic analysis (such as cardiac output) is expected during awake VA-ECMO support. In addition, further studies on the establishment of a disease model in large animals and related pathophysiology exploration are also expected.

## Conclusions

We established a VA-ECMO ovine model by cannulation *via* the jugular vein and artery, which could achieve both respiratory and circulatory support for 7 days. The perioperative management strategies and animal care are the key points of this model. This model could be a platform for further research on disease animal models, hemodynamic analysis, pathophysiology exploration, and new equipment verification.

## Data Availability Statement

The raw data supporting the conclusions of this article will be made available by the authors, without undue reservation.

## Ethics Statement

The animal study was reviewed and approved by Institutional Animal Care and Use Committee (IACUC) of Fuwai Hospital.

## Author Contributions

BJ conceived and originally designed the research and approved the final manuscript. JQ conducted the experiment and wrote the draft of the manuscript. JQ and SG analyzed the data. JQ, SG, WY, and QZ extracted data from the electronic and paper databases. GL, SY, and MZ conducted the experiment and revised the section Discussion of the manuscript. JW, YT, CZ, and QW conducted the experiment and revised the section Materials and Methods of the manuscript. All authors contributed to the article and approved the submitted version.

## Conflict of Interest

The authors declare that the research was conducted in the absence of any commercial or financial relationships that could be construed as a potential conflict of interest.

## Publisher's Note

All claims expressed in this article are solely those of the authors and do not necessarily represent those of their affiliated organizations, or those of the publisher, the editors and the reviewers. Any product that may be evaluated in this article, or claim that may be made by its manufacturer, is not guaranteed or endorsed by the publisher.
